# Understanding Sex‐Specific Behavioral States of Bobcats in Response to Highway Proximity in South Texas

**DOI:** 10.1002/ece3.74079

**Published:** 2026-08-02

**Authors:** Rupesh Maharjan, Sean Kiernan, Jack G. Towson, Elizabeth A. Saldo, Thomas M. Langschied, Emma K. Brookover, Daniel G. Scognamillo, John H. Young, Michael E. Tewes

**Affiliations:** ^1^ Caesar Kleberg Wildlife Research Institute Texas A&M University‐Kingsville Kingsville Texas USA; ^2^ Safari Club International Foundation San Antonio Texas USA; ^3^ Texas Department of Transportation Austin Texas USA

**Keywords:** bobcat, GPS collar data, ocelot, ranchland, road mortality, Texas, wildlife crossing structures, wildlife‐vehicle collisions

## Abstract

Roads are a major source of landscape fragmentation that can influence movement patterns, survival, and behavioral states of carnivore species. Understanding their behavior and response to roads is crucial for developing effective mitigation strategies such as wildlife crossing structures, exclusion fencing, and habitat connectivity planning. We examined the behavioral response of bobcats ( *Lynx rufus* ) from private ranchlands adjacent to US Highway 77 in South Texas. We used GPS collar data to track the movement of 10 bobcats (six males and four females) to quantify fine‐scale movement behavior and space use. We used Hidden Markov Models (HMMs) to predict three behavioral states of each individual movement pattern: State 1 (resting), State 2 (moderately active), and State 3 (traveling). Our model indicated that the interaction of distance to the highway and sex influenced bobcat behavioral state transitions. Male and female bobcats differed in nocturnal movement behavior, with females exhibiting slightly longer mean step length (distance between two consecutive relocations) than males, while turning angles (angle between previous and current displacement) were similar between both sexes during the resting state. Both sexes spent most of their time in the moderately active state (State 2) across all distance to highway classes. This behavior is consistent with foraging, territorial patrolling, and searching for mates during night hours. Although both sexes were moderately active, females reduced movement closer to the highway, whereas males showed more extensive travel. Home ranges of some bobcats overlapped and frequently abutted US Highway 77, suggesting the highway may function as a behavioral and spatial boundary. These findings highlight how expanding highways may reduce functional connectivity for bobcats and other felids and may inform mitigation strategies to reduce wildlife‐vehicle collisions, with potential applications for the conservation of sympatric felids like ocelots (*Leopardus pardalis*).

## 1 Introduction

Many ecosystem features, such as function, structure, and species composition, are strongly impacted by the presence of roads (Coffin 2007). Road impacts are ubiquitous, as approximately 15%–20% of the United States land area is ecologically impacted by roads (Forman and Alexander 1998). One of the major impacts of roads is habitat fragmentation, which reduces landscape connectivity for wildlife and increases mortality risk during road crossings (Reed et al. 1996), especially for endangered species like the ocelot (*Leopardus pardalis*; Haines et al. 2005). Roads may also subtly alter animal behavior by shaping movement and activity patterns (Poessel et al. 2014). Despite growing research in road ecology, the behavioral response of bobcats (*Lynx rufus*) and other meso‐carnivores to roads remains poorly studied in South Texas (Cain et al. 2003; Hanley et al. 2025; Maharjan 2024). Although some studies have examined the effectiveness of wildlife mitigation measures in the region, including wildlife exits (Maharjan, Langbein, et al. 2025), wildlife guards (Maharjan, Young Jr., et al. 2025), and wildlife crossing structures (Roy et al. 2024), the behavioral ecology of bobcats in relation to these features is insufficiently understood. This lack of knowledge limits our ability to design mitigation measures that could reduce the negative impacts of roads on endangered wildlife species (Ng et al. 2004; Poessel et al. 2014).

Roads usually pose a barrier to the movement of wide‐ranging felid species living in habitats fragmented by road networks. For example, mountain lions ( *Puma concolor* ) in southern California avoided paved roads when establishing their home ranges (Dickson and Beier 2002), and Florida panthers (*P. c. coryi*) contracted their home range to avoid crossing roads (Cramer and Portier 2001). These behaviors highlight the effects of roads as a major factor in shaping the home range and territory of these felid species. Additionally, Taylor et al. (2002) found that wildlife‐vehicle collisions (WVCs) result in high mortality rates among individuals that attempt to cross roads, with approximately 35% of wildlife deaths attributable to road mortality. Moreover, anthropogenic disturbance along with the construction of structural features has often decreased suitable felid habitat, increasing the risk of mortality (Burdett et al. 2010; Dickson and Beier 2002; Maharjan et al. 2024; Shrestha, Bishwakarma, et al. 2025).

Bobcats has been one of the wild cat species impacted by the growing road density (Jones et al. 2022; Zheng et al. 2024). Impacts of roads are frequently observed in home ranges of bobcats across the United States (Cramer and Portier 2001; Dickson and Beier 2002; Poessel et al. 2014). Although the species is tolerant to urban environments (Riley et al. 2010), WVCs have become a major source of bobcat mortality in different parts of the USA. These mortalities are common in areas with a high presence of road networks and open crossing spaces (Riley et al. 2003), particularly in South Texas (Maharjan, Young Jr., et al. 2025), Ohio (Bencin et al. 2019), and Southern California (Poessel et al. 2014). Bobcats in Wisconsin had their home ranges in areas with low density of highways, where the frequency of an individual crossing the paved road was lower than expected (Lovallo and Anderson 1996). This suggests that bobcat movement and habitat selection could be widely impacted by roads and traffic. Alternatively, bobcats in the Los Angeles metropolitan area with a high proportion of human‐dominated landscapes had larger home ranges due to the presence of less suitable habitat (Riley et al. 2003). Often, the perimeters of bobcat home ranges bordering major highways or roads were smaller compared to those whose home ranges did not border the roads (Riley et al. 2003). This suggests that roads not only affect their home range size but also alter their home range shape (Riley et al. 2003; Poessel et al. 2014).

Tracking the movements of animals can provide crucial information about their interactions, behavior, and population dynamics (Hooten et al. 2017; Patterson et al. 2017). Their movement patterns can provide a better understanding of foraging behavior, search for mates, predator avoidance mechanisms, and the temporal and spatial extent of dispersal (Wang 2019). Their movement behaviors, including how quickly they moved, how far they traveled, and how tortuous their movement paths were, can indicate how roads impact animal behavior (Shepard et al. 2008; Bennison et al. 2018). The Hidden Markov Model (HMM) has been shown to be a popular method for identifying the behavioral state of animals from their movement patterns. It uses step length (distance between two consecutive relocations) and turning angle (angle between previous and current displacements) as measures to determine individuals' behavioral states. For instance, short step length and slow movement with large turns might indicate animals being in a resting or foraging state, while fast movement with little turning angle and long step lengths might indicate them traveling (Morales et al. 2004). The thorough examination of turning angle and step length can help us study behavioral patterns of animals at a fine spatial scale (Wilson et al. 2008; Nathan et al. 2012). This has been done for species like caribou ( *Rangifer tarandus* ; Franke et al. 2004), gray wolves ( *Canis lupus* ; Ylitalo et al. 2021), Persian leopard ( *Panthera pardus saxicolor* ; Farhadinia et al. 2020), and lion ( *Panthera leo* ; Goodall et al. 2019).

It is crucial to understand the movement behavior of species in response to the landscape features where they live (Morales et al. 2010; Tracey et al. 2013) because their movement influences where they rest, feed, reproduce, and survive. In South Texas, movement in relation to the highway has not been thoroughly studied (Sergeyev et al. 2023), which has resulted in limited information on bobcat behavioral ecology around highways, causing disparity and difficulty in understanding the influence of landscape and environmental features. To address this issue, we applied HMMs to study the movement patterns of bobcats residing within private ranches adjacent to the United States Highway 77 (US77) in South Texas, which is currently undergoing expansion. It has been shown that bobcats are moderately sensitive to habitat fragmentation (Poessel et al. 2014), and its study could be used as an indicator of landscape connectivity (Crooks 2002). To assess this, we focused on (1) understanding how bobcats respond to a highway within their home ranges and (2) identifying if there is a difference in behavioral response based on sex, including home range placement and size. Based on past studies, roads have been shown to alter felid movement and their space use (Sergeyev et al. 2023; Shrestha, Pandey, et al. 2025). So, we expected the bobcat's behavioral state to vary with distance from the highway and between the sex categories. Because males and females may have different spatial requirements, we also expected males to have a larger home range than females.

## 2 Methods

### 2.1 Study Area

**FIGURE 1:**
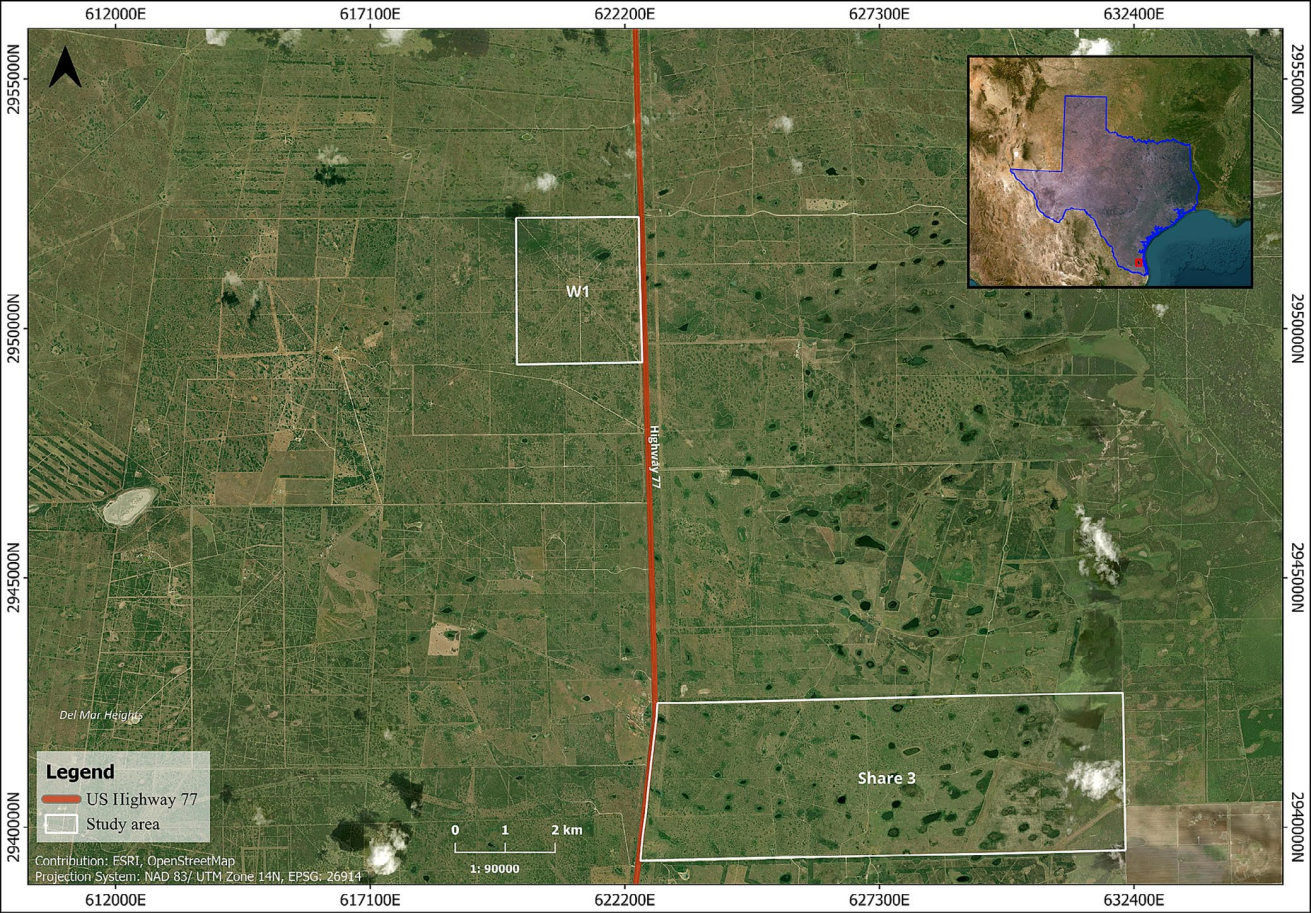
Two study areas adjacent to U.S. Highway 77 in South Texas. Both of the study areas are owned and managed by two separate private entities, that is, W1 and Share 3.

This study was conducted on two privately owned ranches (W1 and Share 3) in southern Texas, located in Kenedy and Willacy Counties, USA. The ranchland lies adjacent to US77, a major north–south highway running from Brownsville, Texas to the central United States with an estimated annual average daily traffic of 16,000 and a speed limit of 120 km/h. It is a four‐lane highway with a vegetated median separating two southbound lanes from two northbound lanes. However, recent expansion projects are converting it into an eight‐lane highway, removing the median vegetation and vegetation along the right‐of‐way. Our study areas are located near each other along the segment of US77 stretching from Raymondville to Sarita and share similar geographic and ecological features (Figure 1). The ranchlands are surrounded by a high cattle fence (~1.2 m) made up of mesh wire on both sides of the highway and have been used for raising cattle and private hunting, suggesting a similar level of habitat structure and human disturbances across both sides. This region of Texas is mostly dominated by honey mesquite ( *Prosopis glandulosa* ), mid‐story (3–5 m) thornscrub forest, and coastal gulf prairie, with varying patch sizes and vegetation density (Jahrsdoerfer and Leslie 1988; Elliott et al. 2014). The area is mostly hot and humid throughout the year, with average temperatures ranging from 10°C in winter (January) to 36°C in summer (July; Palecki et al. 2020). It has variable rainfall patterns ranging from 313 to 529 mm annually with episodic droughts between (Cooper and Wagner 2013).

### 2.2 Bobcat Capturing and GPS Collaring

We captured bobcats using box‐trap wire cages (Tomahawk Live Trap, USA) between January 2025 and April 2025. At W1, we deployed 28 traps for 28 nights (784 trap‐nights) and at Share 3, we deployed 30 traps for 22 nights (660 trap‐nights), resulting in a total trapping effort of 1444 trap‐nights across both study sites. Trap locations were identified based on expert knowledge of bobcat behavior, including signs and tracks. They were placed along potential bobcat movement paths such as wildlife trails, natural drainages, and unpaved roadways through the sites. A grid of traps was placed within 2 miles of US77 on both sides of the highway, with the majority on the west. This was done to evaluate the movement behavior of individual bobcats living close to the highway. Captured bobcats were sedated using a combination of Medetomidine and Ketamine, with dosages based on yearly veterinary recommendations. During a workup, a GPS collar weighing less than 5% of the animal's body weight was fitted (Ryan‐Schofield et al. 2024). Later, Atipamezole was used to reverse the effects of the sedation drugs, and the bobcat was released. Fieldwork in the above procedures followed methods outlined by Beltran and Tewes (1995) and Shindle and Tewes (2000) and was conducted in accordance with protocols approved by the Texas A&M University‐Kingsville Animal Care and Use Committee (protocols #00000020, #00000024, and #2024‐09‐27B). Among 11 bobcats captured, one was fitted with a Telonics TGW 4177‐4 collar with a CR‐7B CTN drop‐off device (Arizona, USA) and 10 with Lotek LifeTrack Iridium 150 collars with ~22‐week drop‐offs (New Zealand). The Telonics collar was programmed to send a GPS fix every hour, and the Lotek collars were programmed to send a GPS fix every 30 min from dusk (6 pm) until dawn (6 am) and once at noon every day. Due to the inconsistent fix rates between the two collar types, we only used data collected between dusk and dawn from the bobcats fitted with Lotek collars in our analyses, omitting the data collected from the Telonics collar.

### 2.3 Data Analysis

GPS collar data from 10 individual bobcats (six male and four female), collected between January and October 2025, were used in the analysis. In post‐processing, collar data were cleaned to maintain positional accuracy by removing or correcting erroneous locations and timestamps. Fixes with missing coordinates, invalid timestamps, or dilution of precision (DOP) values exceeding a defined threshold (DOP > 10) were excluded. Locations with unrealistic movement speeds or displacements exceeding biologically plausible limits for bobcats were identified and removed using step length and temporal filters. To prevent irregular temporal structure, duplicated timestamps and irregular fix intervals were corrected to maintain temporal consistency. All spatial data were projected to the NAD 1983 UTM Zone 14 N coordinate system for subsequent spatial analyses.

### 2.4 Hidden Markov Model

In this study, a Hidden Markov Model (HMM) was used to classify bobcat movement into discrete behavioral states based on GPS collar data. HMMs are time‐series models that infer unobservable behavioral states from observed movement metrics such as step length and turning angle (Hooten et al. 2017). Behavioral states are assumed to follow a first order Markov chain, in which the probability of moving to a new state is influenced by the previous state (Hooten et al. 2017). It is a time‐series model that uses the animal's actual movement (observable time series) and its movement behavior (hidden state sequence) to analyze the time spent on each of the hidden states, which cannot be perceived directly (Shrestha, Pandey, et al. 2025). Those states are influenced by animal behaviors such as resting, foraging, hunting, and traveling. Each behavioral sequence is characterized by the step length and the turning angle. Based on this information, proportional time spent within each behavioral state (activity budgeting) can be obtained from the fitted HMM.

We evaluated both 2‐State (resting and traveling) and 3‐State models (resting, moderately active, and traveling) with and without distance to highway as a continuous covariate. In the 2‐State movement model, State 1 was characterized as shorter and undirected step length (resting phase) while State 2 was marked as longer and directed step length (traveling phase). The 3‐State model anticipated three behavioral states, where State 1 is characterized as short step length with high turning angle (close to −π to π). This state was represented as a slow and indirect movement of wildlife during the resting phase. State 2 was expected to have low turning angles and relatively moderate step length, indicating moderate active movement. Animals in State 3 indicated fast and directed movement, associated with the traveling state, and were expected to have longer step length and a small turning angle. However, upon comparison, the 3‐State model consistently received more AIC support; all subsequent analyses were based on the 3‐State model (Table S1).

An HMM requires time series data in discrete form to fit the model. The model from HMM is fitted by numerical maximization and generated from the likelihood function, which requires an initial set of parameters to start the iterative optimization process (Michelot and Langrock 2023). Those parameters are selected based on exploratory data analysis, where a histogram of step length and turning angle is used to derive state‐specific means. For step length, the observed mean and standard deviation are used, while the turning angle uses the observed mean and inverse variance to initialize a 2‐State or 3‐State behavioral model (Michelot and Langrock 2023). Hence, these parameter estimates are used to generate a likelihood estimate of the observed data, enabling the identification of all possible hidden state sequences, adequately representing the observed temporal patterns (Pohle et al. 2017; Patterson et al. 2019).

To carry out HMM analysis, the data point has to be continuous, so we first ordered locations by time and then calculated the time interval between each successive fix. This allowed us to partition the data into nightly trajectories, referred to as “bursts,” which are defined as contiguous sequences of locations separated by the start of a new night. Each burst was treated as a separate, or independent sequence, to fit into the HMM analysis. The step length and turning angles for each step were calculated using Universal Transverse Mercator (UTM) coordinates through the *momentuHMM* package (Version 4.4.2; McClintock and Michelot 2018) in R. We fitted a 2‐State and 3‐State model to analyze the behavioral patterns of each bobcat using HMMs with Wrapped Cauchy turning angles and gamma‐distributed step lengths (Mastrantonio 2015). Transition probabilities between states in relation to distance to the highway (US77), sex, and interaction between sex and distance (sex × distance to highway) were used as covariates in the model. The logit of transition probabilities for each transition is modeled as:

$$\begin{array}{c} \text{logit}\;P_{\mathrm{ij}}=\beta_{0}+\beta_{1}\;\text{SexMale}+\beta_{2}\;\text{distance to highway}\\ \;+\beta_{3}\;\left(\text{SexMale}\times \text{distance to highway}\right) \end{array}$$

where

$$\text{Female}\;\left(\text{SexMale}=0\right):\text{logit}\;P_{\mathrm{ij}}=\beta_{0}=\beta_{2}\times \text{distance to highway}$$

$$\begin{array}{c} \text{Male}\;\left(\text{SexMale}=1\right):\text{logit}\;P_{\mathrm{ij}}=\left(\beta_{0}+\beta_{1}\right)+\left(\beta_{2}+\beta_{3}\right)\\ \;\times \text{distance to highway} \end{array}$$

This regression coefficient describes how distance to the highway and sex jointly affect the transition between behavioral states. The model selection was done using AIC, comparing the null model, the distance to highway only model, the additive model with main effects of distance to highway and sex, and a model with sex × distance to highway interaction. The best model was selected, having lower AIC values, which was used to derive the most likely state sequence using the Viterbi algorithm to classify each nocturnal step.

### 2.5 Home Range Data Analysis

Home range estimation encompasses methods such as point, density, and movement‐based approaches, including kernel density estimation and minimum convex polygon methods, which together provide a robust estimate of space use (Bevanda et al. 2016). Various methods have effectively been used to estimate home ranges of several felid species, including bobcats. In this study, the home range for each individual was estimated using a minimum convex polygon (MCP), and kernel density estimation (KDE) with 95% and 50% isopleths calculated in R (Version 4.4.2) using the *adehabitatHR* package (Calenge 2011). For each bobcat, we estimated kernel utilization distribution (KDE, href bandwidth) and derived 50% and 95% isopleths representing their overall core use and home range, respectively. For mapping, we show the 95% KDE to represent the extent of the nocturnal home range and the 50% KDE to highlight core activity areas. Because KDE represents the underlying utilization distribution and is less influenced by outliers, we use KDE as our primary home range metric and report on MCP for comparison (Seaman and Powell 1996).

## 3 Results

Among 10 bobcats that were GPS‐collared in two private ranchlands, three individuals were captured in Share 3, and the remaining seven were in the W1 study site. Among the three captured bobcats in Share 3, two were male, and one was female. In W1, four were males and three were females (Table 1). The highest number of fixes belonged to individual SH3B01M, with 279 days of fixes, followed by SH3B04M with 229 days. After pre‐processing, there was a total of 14,834 GPS fixes across all 10 individuals, among which bobcat SH3B01M had 3030 GPS fixes, while individual W1B08M had the lowest number of locations with only 189 GPS fixes (Table 1).

**TABLE 1:** Information on individual bobcats captured in “Share 3” and “W1” study sites located in Kenedy and Willacy Counties, Texas, including their demographic and physiological characteristics and capture details.

Location	Name/ID	Sex/age	Body mass (kg)	Capture date	Last fix date	Number of days	Number of location points
Share 3	SH3B01M	M	9.20	1/15/2025	10/21/2025	279	3030
Share 3	SH3B02F	F	6.51	2/13/2025	9/19/2025	218	2630
Share 3	SH3B04M	M	10.02	3/7/2025	10/22/2025	229	2272
W1	W1B01M	M	11.10	3/26/2025	8/25/2025	152	1541
W1	W1B02F	F	8.16	3/26/2025	10/18/2025	206	1683
W1	W1B03F	F	9.55	4/7/2025	6/19/2025	73	476
W1	W1B04M	M	9.49	4/5/2025	5/31/2025	56	318
W1	W1B05M	M	10.38	4/11/2025	9/10/2025	152	1271
W1	W1B06F	F	8.45	4/27/2025	9/12/2025	138	1424
W1	W1B08M	M	8.88	4/14/2025	5/12/2025	28	189

### 3.1 Movement Behavior

Among all the HMMs, the 3‐State model including sex × distance to highway had the substantially lowest AIC and ΔAIC value (Table 2), indicating that behavioral state transitions were influenced by sex and proximity to the highway. However, models with a single covariate had a lower ability to discriminate between different behavioral states (Table 2). Estimating the 3‐state movement behavior of 10 bobcats revealed distinct movement patterns among different states (Table 3). Both males and females showed slightly different nocturnal movement states, where females displayed short steps (mean ± SD: 9.5 m ± 7.2), and intermediate turning angle concentration (*k* = 0.18), and males showed slightly shorter steps (8.9 m ± 6.2) but similar angular concentration (*k* = 0.21) compared to females in the resting state. In a moderately active state, females had shorter step length (143 m ± 105) and relatively lower angle of concentration (*k* = 0.45) compared to males (mean step length = 205 m ± 128; *k* = 0.56), indicating directed movement over moderate distances. However, males had lower step length (689 m ± 621) and angle of concentration (*k* = 0.15) compared to females (833 m ± 617; *k* = 0.22) in the traveling state, indicating long‐distance and less constrained travel (Table 3). Activity budget estimates showed that males spent a smaller fraction of time resting (0.18; CI: 0.167–0.183; SE: ±0.004) and displayed a somewhat larger proportion traveling (0.11; CI: 0.104–0.117; SE: ±0.003) compared to females (resting: 0.25; CI: 0.236–0.257; SE: ±0.005; traveling: 0.04; CI: 0.040–0.050; SE: ±0.003) while maintaining moderately active nocturnal budget state by both sexes (Table 3).

**TABLE 2:** Akaike information criterion (AIC) based comparison of candidate Hidden Markov Models fitted to bobcat movement data in South Texas, 2025.

Model	*k*	logLik	AIC	ΔAIC
3‐State model (sex × distance to highway)	41	−114,460	229,002.6	0
3‐State model (sex + distance to highway)	35	−114,477	229,024.2	21.64815
3‐State model (sex)	29	−114,506	229,069.3	66.72552
3‐State model (distance to highway)	29	−114,522	229,101.2	98.56032
3‐State model (Null)	23	−114,557	229,160.1	157.5452

**TABLE 3:** State‐dependent movement characteristics (step length, turning angle concentration) and activity budgets for male and female bobcats.

	State 1 (resting)	State 2 (moderately active)	State 3 (traveling)
Female			
Step mean	9.52	143.21	832.82
Step SD	7.20	105.45	616.80
Angle mean	−2.99	−0.01	2.78
Angle concentration	0.18	0.45	0.22
Activity budget	0.25 (CI: 0.236–0.257 ± 0.005)	0.71 (CI: 0.697–0.720 ± 0.006)	0.04 (CI: 0.040–0.050 ± 0.003)
Male			
Step mean	8.85	204.72	688.64
Step SD	6.20	128.30	621.40
Angle mean	3.05	0.02	−3.03
Angle concentration	0.21	0.56	0.15
Activity budget	0.18 (CI: 0.167–0.183 ± 0.004)	0.71 (CI: 0.705–0.725 ± 0.005)	0.11 (CI: 0.104–0.117 ± 0.003)

*Note:* The step mean indicates the average distance between two consecutive GPS fixes in a given state, where the higher the step mean, the higher the state bobcats are in. Step standard deviation (SD) indicates variation in step length within that state, and Angle mean explains the change in direction between consecutive steps. Angle concentration shows how tightly angles are clustered around the mean angle, and activity budget is the proportion of time that the bobcat spends in each hidden state.

**FIGURE 2:**
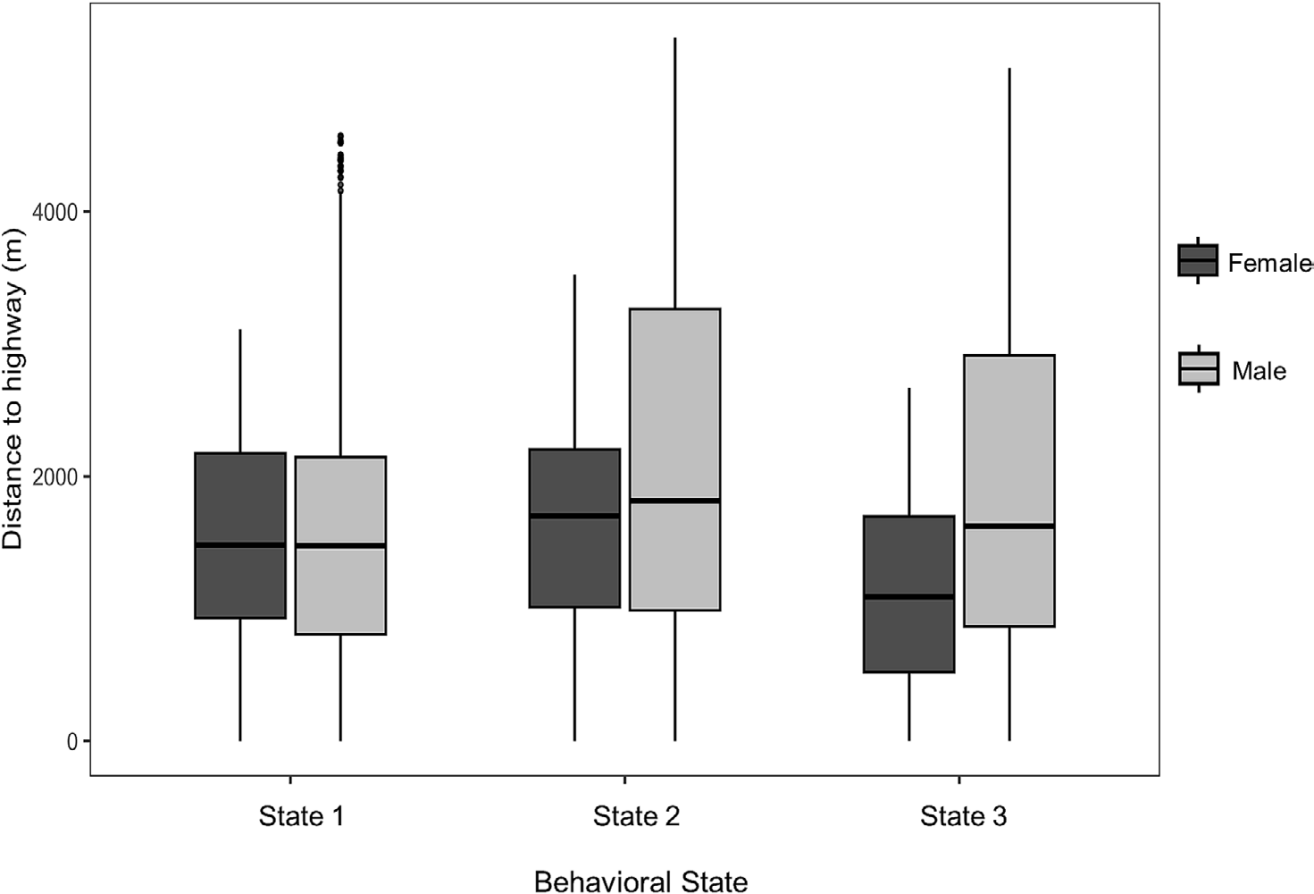
Comparison of three behavioral states (State 1, State 2, State 3) by distance to the highway in meters and sex of bobcats in South Texas, 2025. States 1, 2, and 3 represent resting, moderately active, and traveling phase respectively.

**FIGURE 3:**
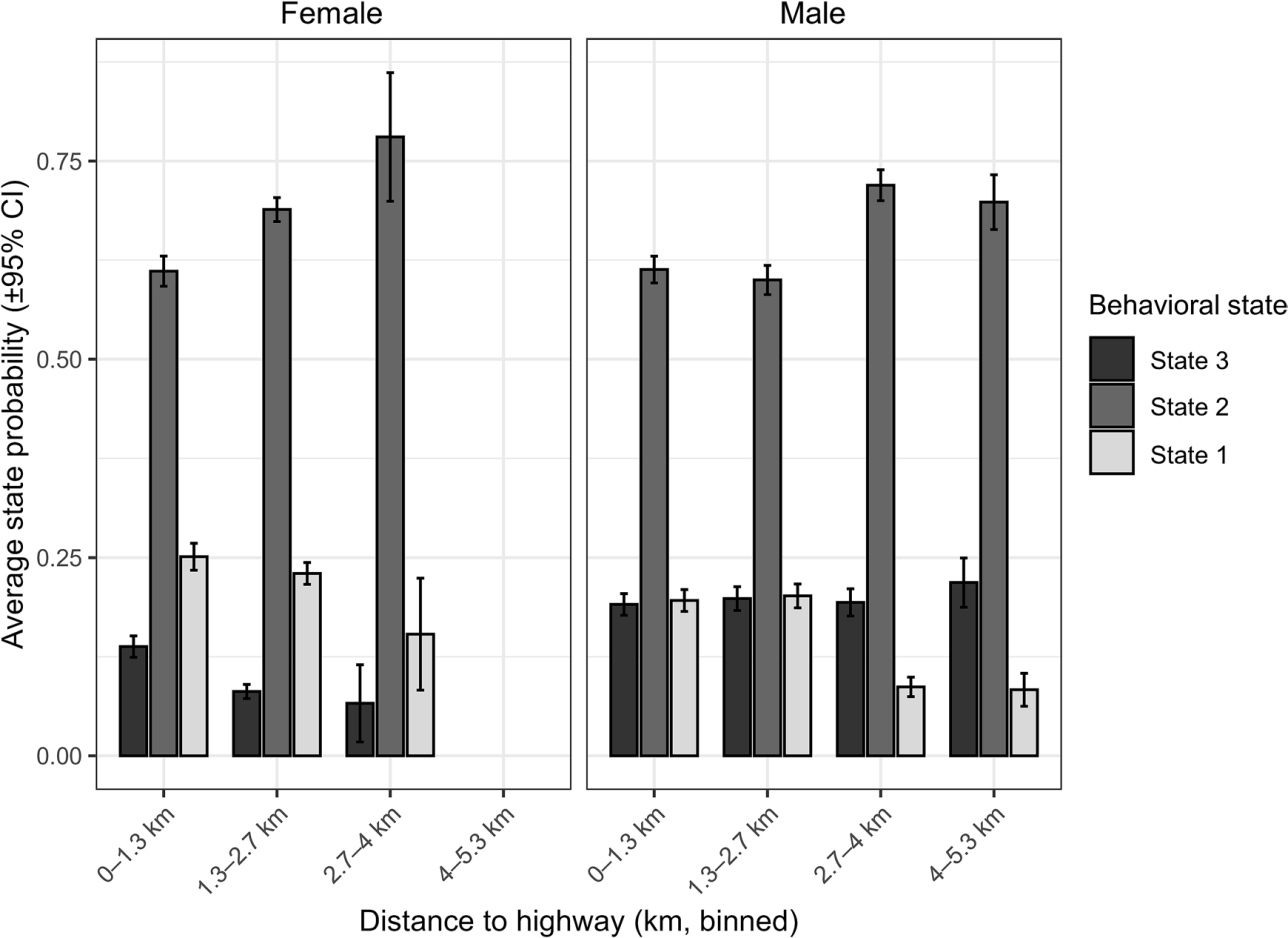
Sex‐specific differences in average behavioral state probabilities across distance to highway bins in bobcats. States 1, 2, and 3 represent resting, moderately active, and traveling phases respectively.

The spatial placement of behavior by two different sexes (male and female) was influenced by state‐specific distances (Figures 2 and 3), where traveling steps (State 3) for females occurred closer to the highway (mean distance: 1.15 km; SD: ±746 m) compared to resting (State 1; 1.5 km; SD: ±770 m) and moderately active (State 2; 1.6 km; SD: ±738 m) states. This suggests that closer to the road corridor, the concentration of high‐displacement movement is also higher for females. However, males tend to rest closer to the road (State 1; 1.6 km; SD: ±1125 m) and have higher traveling (State 3; 1.9 km; SD: ±1248 m) and moderately active (State 2; 2.07 km; SD: ±1293 m) states farther from the highway corridor, indicating that males display more active movement states farther from the highway. Similarly, the binned state probabilities show responses in which the behavioral state varies with distance to the highway and sex. For both males and females, the moderately active state dominated across all distance classes compared to the other two states, with an increase in average state probability as distance from the highway increased (Figure 3). However, the average state probability for the resting state in both sexes decreases with an increase in distance.

**FIGURE 4:**
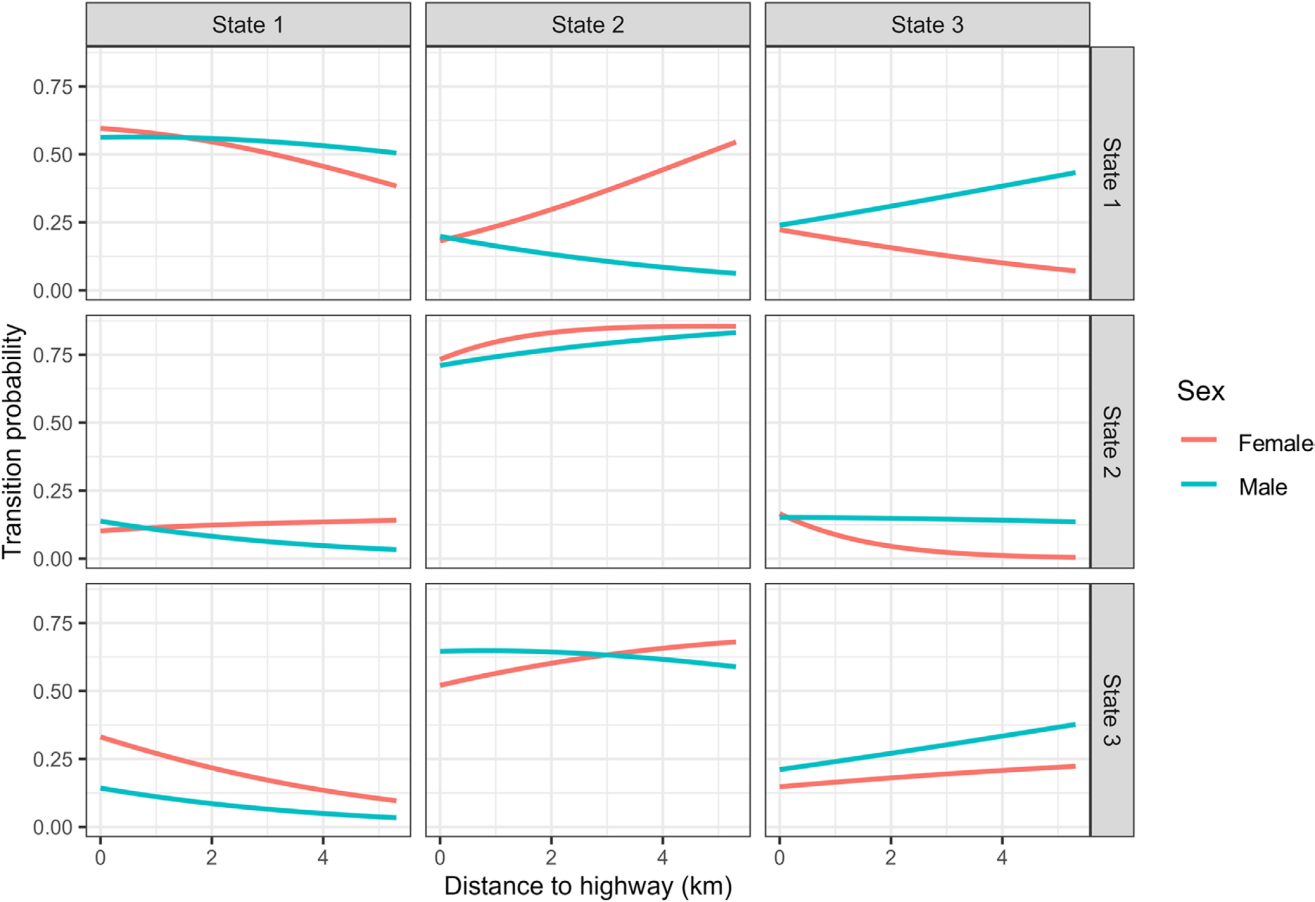
Predicted transition probability among three behavioral states for male and female bobcats across distance to highway gradients.

The transition probability matrix for the best supported model showed several clear sex‐specific road response transitions. For the transition from State 1 to State 2, there is a positive distance coefficient for females (*β*_1_ → *β*_2_, distance to highway = +0.32; Table S2), indicating that there is a higher probability of female bobcats switching from State 1 to State 2 as they move farther from the road (Figure 4).

### 3.2 Home Range

**FIGURE 5:**
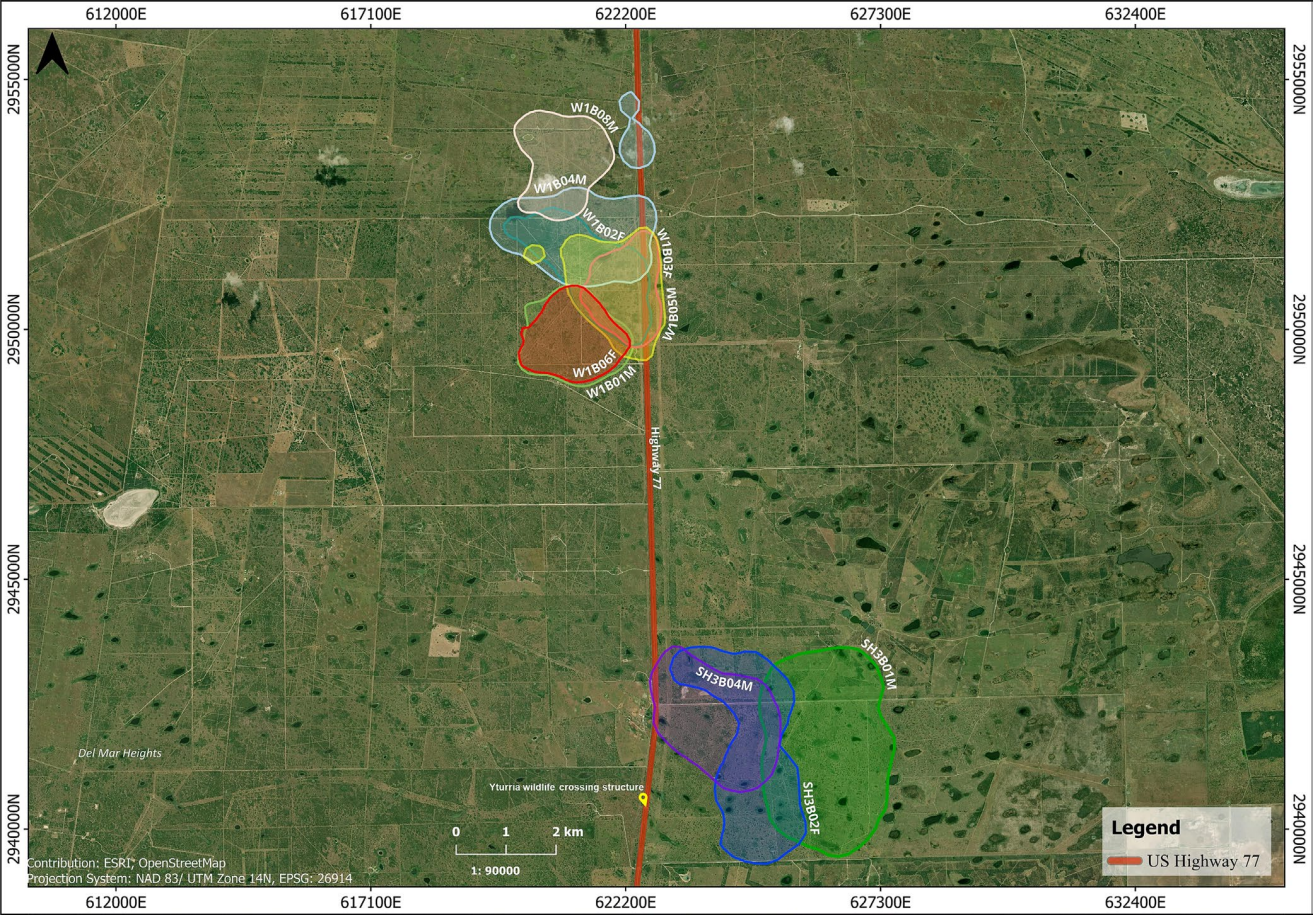
Estimated 95% kernel density (KDE) home ranges for 10 bobcats in the W1 and Share 3 study areas of South Texas, located adjacent to U.S. Highway 77.

The home ranges of all GPS‐collared bobcats varied from 1.81 to 7.97 km^2^ at 95% and 0.59 to 3.79 km^2^ at 50% estimated by MCP, while in KDE href from 2.58 to 9.07 km^2^ at 95% and 0.78 to 3.30 km^2^ at 50%. The estimates produced by KDE href are comparatively higher than the estimates produced by MCP (Table 4). The home range core area is represented by the 50% isopleth area, which is two to three times smaller than the 95% isopleth area for each individual. Among 10 individuals, SH3B01M (Male) had the largest home range (95% = 7.97, 50% = 3.79) based on MCP, while W1B08M had the smallest home range (95% = 1.81, 50% = 0.59). There was a high level of home range overlap seen in the bobcats from W1 (Figure 5) compared to those from Share 3. Although most of the bobcats did not cross the highway frequently, several individuals (W1B02F, W1B05M, W1B03F, W1B04M) ventured across the highway, a very short distance, before concentrating their movement in the west.

**TABLE 4:** Home range estimates for 10 bobcats based on Minimum Convex Polygon (MCP) and Kernel Density Estimation (KDE) methods, showing 95% and 50% isopleths.

Name/ID	MCP (km^2^)		KDE href (km^2^)	
	95%	50%	95%	50%
SH3B01M	7.97	3.79	9.07	3.30
SH3B02F	6.76	1.50	6.38	0.99
SH3B04M	4.12	1.64	5.15	1.59
W1B01M	2.66	1.23	3.30	1.16
W1B02F	3.16	0.98	3.98	1.02
W1B03F	1.85	0.75	2.58	0.78
W1B04M	2.67	0.87	5.52	1.23
W1B05M	2.92	1.22	4.01	1.29
W1B06F	2.13	0.92	2.88	0.97
W1B08M	1.81	0.59	3.11	0.83
Mean size	3.60	1.35	4.60	1.32

## 4 Discussion

Our results demonstrate that bobcat movement is best explained by the interaction between distance to highway and sex covariates, resulting in resting, moderately active, and traveling states. There was variation in both step length and turning angle between the two different sex categories. Males exhibited higher step length with lower turning angles during the active state (moderately active and traveling) compared to females. This may indicate that male and female bobcats use different strategies for resource allocation or utilization. Although a study on bobcats showed that both males and females displayed increased movement patterns during their active state (McNitt et al. 2020), the bobcats in our study displayed varied directed movement between sexes, with females moving over a moderate distance compared to males. Further, our HMM results suggest a strong interaction between distance to highway and sex groups in bobcats. Although Sergeyev et al. (2023) and Kirby et al. (2010) found the relationship between time of day and activity pattern to be a significant predictor of bobcat space use, we did not examine this because our datasets lacked daytime data. In addition, studies have found other environmental covariates such as slope, elevation, aspect, and ruggedness as significant factors influencing the home range and resource selection strategies (Rosenbaum et al. 2023). We did not include these factors because South Texas is mostly characterized by relatively flat terrain, with minimal variation in slope, elevation, and aspect. Also, we did not use vegetation and distance to water as covariates as our focus was mainly to see the influence of highway distance on the movement behavioral state of bobcats. Our objective was to conduct a focused test of whether proximity to a highway is associated with transitions between movement states in bobcats. Therefore, we specified a parsimonious model including only biologically motivated predictors directly related to two of our covariates (distance to highway and sex).

Our study found that males reduced their activity patterns closer to the highway and displayed higher activeness farther from it. This pattern is consistent with findings from Poessel et al. (2014) showing that male bobcats occupying areas closer to highways tend to have smaller home ranges and reduced movement, whereas those farther from roads maintain larger home ranges and display increased activity. Both male and female bobcats spend most of their time in a moderately active state across all distance classes compared to the other two states. When individuals remain longer in the moderately active state, it indicates that they are actively moving for hunting, patrolling their home range, or searching for mates (Grönberg 2011; Krofel et al. 2021). We found that males are less likely to switch their movement behavior from resting to a moderately active state, which might be due to disturbance and fragmentation closer to the road, forcing them to adjust their behavior frequently. Also, it could be due to abrupt changes in cover and prey activity in areas close to the road, resulting in more alternating behavior.

The estimated home range closer to the road was similar to the home ranges estimated by a previous study in South Texas (Leonard 2016). This study addressed the home ranges of bobcats on ranches ranging from 10 to 40 km^2^ for males and 5 to 12 km^2^ for females. Our study found that the average home range for male bobcats ranged from 3.6 to 5.02 km^2^ and ~3.4 to 3.9 km^2^ for females, using 95% MCP and KDE. The home ranges of KDE were generally smaller and more focused around areas of intensive use, whereas MCPs tended to be larger and more sensitive to occasional long‐distance excursions. In addition, the home ranges of some bobcats overlapped with each other, and only a few individuals had a home range extending across the highway. However, examining their movement behaviors, almost all of them used the highway as their boundary. This behavior has also been documented by Poessel et al. (2014) and Riley et al. (2006), in which 90% of bobcats did not cross the road and treated it as a boundary of their home range. This suggests that bobcats often avoid high‐traffic roads and tend to restrict their movement along the road rather than crossing it frequently. We found that one male individual remained in the vegetated median in US Highway 77 for a long period of time but rarely crossed the highway.

Although most bobcats avoided the highway in our study, they could be more likely to cross the road during resource scarcity and competition in the future, forcing the weak ones to abandon their territory. This will likely result in higher bobcat mortality risk while crossing the highway (Riley et al. 2003). Despite the presence of a major bridge‐style wildlife crossing structure and the closer proximity of bobcat home ranges to it, none of the GPS‐collared bobcats used the structure during the study period. This may be because the existing crossing structure was located more than 1.6 km from Share 3 ranch, where most of the monitored bobcats maintained their home ranges. We observed that both sexes were moderately active; however, females were more cautious and limited their movement close to the road compared to males. This type of behavior was also documented by Poessel et al. (2014) and Bencin et al. (2019), in which bobcats limited but did not avoid their movement close to highways. This suggests bobcats establish their home range in a way that they either avoid or minimize crossing busy roads.

Bobcats often exhibit overlapping home ranges depending on habitat conditions, relatedness among individuals, and spatial organization within the population (Nielsen and Woolf 2001; Young et al. 2019). Our study found that the home ranges of some females (*n* = 4; SH3B02F, W1B02F, W1B06F, 21B03F) overlap with those of males or other females (Figure 5). This is not very unusual in feline species, as subadult females tend to overlap their home ranges with their mothers (Johansson et al. 2024; Payne et al. 2025). Studies have found that adults often prioritize their home range size based on the need for resources for themselves and their young (Johansson et al. 2018). This behavior has been observed in ocelots, where the home range size changes with reproductive needs, and females may expand their space use when energetic demand increases during breeding season (Smith et al. 2025). However, we did not evaluate differences in seasonal or reproductive‐period movement behavior due to variation in capture date and monitoring duration among individuals. Also, we were unable to confirm the reproductive status, denning behavior, and presence of kittens in female bobcats, making seasonal comparisons unreliable and speculative.

Although we did not include vegetation cover as a covariate, the visualization of the collared bobcat states on the map shows that they have been using dense vegetation cover closer to the road while resting, similar to Sergeyev et al. (2023), where the bobcat's resting state was higher close to woody vegetation cover. For females, resting and traveling state decreases with an increase in distance to the highway. This could be because roadside environments often provide dense cover and elevated prey availability, which may attract females with smaller home ranges. Also, it could be due to the presence of denning areas closer to the roads, making their movement more concentrated towards it, or a bobcat might be patrolling its territory during the night and displaying its exploratory behaviors (Karelus et al. 2019; Farhadinia et al. 2020).

Our study has a few limitations that should be considered when interpreting the results and guiding future research. The data collected from the GPS collars were analyzed based on only 10 individual bobcats from a single highway corridor collected within a single field season. So, a lack of reference population or control group may have limited our ability to isolate the highway effect from broader environmental influences. Although our temporal and sample size constraints may limit the ability to generalize our findings to a larger population and wider environmental conditions, the datasets offer crucial information about the fine‐scale movement response of bobcats close to road infrastructure. Most importantly, our study was designed to examine the influence of distance to highway and the sex category on bobcat movement behavior. Therefore, the model was purposefully limited to these predictors and excluded other environmental parameters such as temperature, water source, traffic volume, food, cover, competition, and vegetation composition. Because these factors may also affect bobcat movement and habitat use, we acknowledge that the observed movement patterns might not be caused exclusively by the highway; rather the highway should be interpreted as a potential factor influencing their movement behavior. We also acknowledge that variation in monitoring periods among individuals may have influenced their home range and core area estimates, with estimates for individuals tracked for shorter periods likely to be underestimated. Therefore, these home range estimates should be interpreted as observed space use during the monitoring period rather than precise estimates of population‐level home range size.

We suggest that future studies should emphasize incorporating a control group of bobcats located farther from highways, shorter fix intervals, larger sample sizes, multiple road systems, longer monitoring periods, and additional environmental variables to allow clearer separation of highway‐related behavior response and refine our perspective on how roads interact with landscape features to influence felid movement. Also, the behavioral state of bobcats within the highway corridor could be influenced by seasonal and reproductive variation, so studies with longer, more consistent monitoring periods and knowledge of their reproductive status could help evaluate these effects on their behavior. Nevertheless, the consistent behavioral patterns observed across individuals suggest that even relatively low‐volume highways, such as US Highway 77, may influence bobcat movement and highlight the importance of continued research on road impacts on felid ecology. So, incorporating bobcat telemetry into long‐term road‐monitoring efforts could help transportation agencies evaluate mitigation effectiveness over time.

## 5 Conclusion

In South Texas, the major US Highway 77 appears to function as a boundary for bobcat home ranges. Such linear infrastructures can influence the spatial organization of felids by limiting movement across the landscape and increasing perceived risk of crossing the highway. While few bobcats crossed the road, most of them tend to stay within their home range on one side of the road. Males and females exhibited distinct nocturnal movement strategies, with females reducing movement closer to the highway and males showing more extensive travel, suggesting a stronger effect of highways on female bobcat movements. While these findings are specific to bobcats, they may provide a valuable indicator of the landscape connectivity in southern Texas for endangered species such as the ocelot. This is because bobcats share similar ecological and behavioral traits, such as activity patterns, diet, and habitat use, and are more common and easier to monitor compared to ocelots, particularly in South Texas. As highway expansion continues, these behavioral responses suggest that functional connectivity may be further reduced within bobcats and other felid populations living near highways and have an effect on survival and genetic connectivity. Understanding movement state response to roads provides valuable guidance for transportation planning and wildlife mitigation aimed at maintaining connectivity in increasingly fragmented landscapes.

## Author Contributions

**Rupesh Maharjan:** conceptualization (lead), data curation (equal), formal analysis (lead), investigation (equal), methodology (lead), resources (equal), validation (equal), visualization (equal), writing – original draft (lead), writing – review and editing (equal). **Sean Kiernan:** data curation (equal), methodology (equal), validation (equal), visualization (equal), writing – original draft (supporting), writing – review and editing (equal). **Jack G. Towson:** methodology (supporting), validation (equal), visualization (supporting), writing – review and editing (supporting). **Elizabeth A. Saldo:** conceptualization (equal), methodology (equal), resources (equal), validation (equal), visualization (equal), writing – review and editing (equal). **Thomas M. Langschied:** methodology (supporting), validation (supporting), visualization (equal), writing – review and editing (supporting). **Emma K. Brookover:** data curation (equal), methodology (equal), validation (equal), visualization (equal), writing – original draft (supporting), writing – review and editing (equal). **Daniel G. Scognamillo:** conceptualization (equal), data curation (equal), methodology (equal), project administration (equal), resources (equal), supervision (equal), validation (equal), visualization (equal), writing – review and editing (equal). **John H. Young:** conceptualization (equal), investigation (equal), methodology (equal), project administration (equal), resources (equal), supervision (equal), validation (equal), visualization (equal), writing – review and editing (equal). **Michael E. Tewes:** conceptualization (equal), data curation (equal), formal analysis (equal), funding acquisition (equal), methodology (equal), project administration (equal), resources (equal), supervision (equal), validation (equal), visualization (equal), writing – review and editing (equal).

## Funding

The work was funded by the Texas Department of Transportation and Tim and Karen Hixon Foundation.

## Conflicts of Interest

The authors declare no conflicts of interest.

## Supporting information


**Table S1:** Akaike information criterion (AIC) based comparison of candidate Hidden Markov Models fitted between 2‐State and 3‐State models to bobcat movement data in South Texas, 2025.
**Table S2:** Estimated coefficients for covariate effects (sex, distance to highway, and their interaction) on Hidden Markov Model State transition probabilities between behavioral states (i → j) of bobcat movement.

## Data Availability

Code and a sample of the data that we used for modeling are available in Dryad (Maharjan et al. 2026; https://doi.org/10.5061/dryad.41ns1rnw5).
