# Supplementary material for: Understanding Sex‐Specific Behavioral States of Bobcats in Response to Highway Proximity in South Texas
Source: Ecol Evol. 2026 Aug 2;16(8):e74079. doi: 10.1002/ece3.74079 (PMC13429940; doi:10.1002/ece3.74079)
Supplement: Supplementary file 1 — Table S1: Akaike information criterion (AIC) based comparison of candidate Hidden Markov Models fitted between 2‐State and 3‐State models to bobcat movement data in South Texas, 2025. Table S2: Estimated coefficients for covariate effects (sex, distance to highway, and their interaction) on Hidden Markov Model State transition probabilities between behavioral states (i → j) of bobcat movement. [file ECE3-16-e74079-s001.docx]

**Supplementary**

**Supplementary Table 1.** Akaike Information Criterion (AIC) based comparison of candidate Hidden Markov Models fitted between 2- State and 3- State models to bobcat movement data in South Texas, 2025.

| Model | k | logLik | AIC | ΔAIC |
| --- | --- | --- | --- | --- |
| 3-State | 23 | -114557.1 | 229160.1 | 0.000 |
| 2-State | 13 | -115434.2 | 230894.5 | 1734.341 |

**Supplementary Table 2.** Estimated coefficients for covariate effects (sex, distance to highway, and their interaction) on Hidden Markov Model State transition probabilities between behavioral states (i → j) of bobcat movement.

|  | 1 → 2 | 1 → 3 | 2 → 1 | 2 → 3 | 3 → 1 |
| --- | --- | --- | --- | --- | --- |
| Intercept | -0.67 | -1.22 | -1.92 | -2.76 | 0.25 |
| Sex (Male) | -0.73 | 0.60 | -0.26 | 1.12 | -1.32 |
| Distance to highway | 0.32 | -0.14 | 0.03 | -0.79 | -0.34 |
| Sex × Distance to highway | -0.54 | 0.29 | -0.38 | 0.73 | -0.08 |
